# Evaluating Adherence to the 2023 Canadian Thoracic Society Chronic Obstructive Pulmonary Disease Pharmacotherapy Guidelines: A Hospital-Based Study

**DOI:** 10.1177/29768675251336660

**Published:** 2025-04-30

**Authors:** Mathieu D. Saint-Pierre

**Affiliations:** 12365 University of Ottawa, Faculty of Medicine, Ottawa, ON, Canada; 551435 Institut du Savoir Montfort, Ottawa, ON, Canada; Division of Respirology, 153164 Montfort Hospital, Ottawa, ON, Canada

**Keywords:** chronic obstructive pulmonary disease, guidelines, adherence, inhaled therapy, Canadian Thoracic Society

## Abstract

**Background:**

A previous study at Montfort Hospital (Ottawa, Ontario, Canada) found that only one-fifth of patients treated in 2022 for a severe acute exacerbation of chronic obstructive pulmonary disease (AECOPD) were prescribed the appropriate inhaled therapy at discharge. The revised 2023 Canadian Thoracic Society (CTS) COPD pharmacotherapy guidelines now recommend inhaled triple therapy as initial maintenance treatment in patients at high risk of AECOPD.

**Objectives:**

The primary objective of this study was to determine if adherence to the CTS guidelines significantly improved following the publication of the 2023 statement. A secondary objective was to review the proportion of patients receiving appropriate optimization based on whether they were treated exclusively in the emergency department (ED) or required hospitalization.

**Design:**

Retrospective study

**Methods:**

Subjects treated for AECOPD in the first 12 months after the publication of the 2023 guidelines were reviewed. Patient characteristics and inhaled therapy were charted. Adherence to the guidelines was compared to the prior cohort from 2022.

**Results:**

A total of 169 patients were treated for AECOPD. After excluding individuals who died in the hospital and those who were maintained on inhaled triple therapy, 74 were candidates for review of their inhaled therapy. 27% received recommended medication optimization at discharge compared to 20% in 2022 (P = 0.25). Adherence to the guidelines significantly improved for hospitalized patients (51% vs 27%, P = 0.02). Only 5% of subjects treated exclusively in the ED received appropriate inhaler optimization. The most common deviations from the guidelines were the continued use of prior therapy (35%) and the lack of any long-acting medication (22%).

**Conclusions:**

Adherence to the CTS COPD pharmacotherapy guidelines remained very low in ED-treated patients. The findings highlight the need for structured COPD care plans.

## Introduction

Acute exacerbation(s) of chronic obstructive pulmonary disease (AECOPD) are the leading cause of hospitalization in Canada, after childbirth.^1^ The national cost for AECOPD-related hospitalizations exceeds 4 billion Canadian dollars annually.^2^ The 2019 Canadian Thoracic Society (CTS) COPD pharmacotherapy guidelines (section <<*Preventing acute exacerbations in stable COPD patients*>>) previously recommended for patients at high risk of AECOPD, defined as a history of severe AECOPD (resulting in emergency department (ED) visit or hospitalization) or two or more moderate AECOPD (necessitating oral corticosteroid use and/or antibiotic) in the prior year, an inhaled long-acting muscarinic antagonist (LAMA)/long-acting beta-agonist (LABA) combination as the initial choice of maintenance medication except in subjects with a blood eosinophil count (BEC) ≥ 0.3 × 109/litre (L) where an inhaled corticosteroid (ICS)/LABA was favored.^3^ The revised CTS pharmacotherapy guidelines were published in September 2023. The updated guidelines (sections <<*Preventing AECOPD*>> and <<*Reducing mortality*>>) now recommend inhaled LAMA/LABA/ICS as initial maintenance therapy in patients at high risk of AECOPD, preferably administered in a single inhaler.^4^ This recommendation reflects the greater benefits of inhaled triple therapy (LAMA/LAMA/ICS) compared to dual therapies in reducing the risk of AECOPD and all-cause mortality, in addition to improving health status and lung function.^5,6^

A previous study examined real-world adherence to the 2019 CTS COPD pharmacotherapy guidelines in patients treated for severe AECOPD at Montfort Hospital (Ottawa, Ontario, Canada). This review found that only 20% of patients (22/111) treated in 2022 were prescribed the recommended inhaled therapy, and the lack of appropriate optimization led to a higher rate of readmission for AECOPD within 30 days. The most common deviations from the guidelines were patients continuing their prior long-acting therapy (48%) and the absence of any prescribed long-acting inhaled therapy (18%).^7^

It would seem reasonable to suspect that the simplified treatment recommendation for severe AECOPD patients would lead to improved observance of the guidelines. Therefore, the primary objective of this study was to determine if adherence to the CTS COPD pharmacotherapy guidelines significantly improved following the publication of the 2023 statement. A secondary objective was to review the proportion of patients receiving appropriate optimization based on whether they were treated exclusively in the ED or required hospitalization.

## Materials and Methods

Subjects admitted to the ED or an inpatient unit at Montfort Hospital for a diagnosis of AECOPD in the first 12 months after the publication of the 2023 CTS COPD pharmacotherapy guidelines (October 2023 to September 2024) were retrospectively reviewed (Montfort Hospital research ethics board file #22-23-02-052; participant informed consent was not required as this was a retrospective study). Montfort Hospital is an academic teaching hospital affiliated with the University of Ottawa that provides acute care to approximately 1.5 million people. Lists of patients treated for AECOPD were obtained from the medical archives, and each chart was reviewed to ensure that this was the most likely cause for presentation to the hospital. Patient mortality was also recorded.

Demographic data charted included age, sex, and cigarette smoking status. Home supplemental oxygen use and past spirometry results were examined. BEC available from the prior 12 months at Montfort Hospital were documented. Long-acting inhaled medications (and the number of delivery devices) were recorded at admission for AECOPD (via community pharmacy medication reconciliation) and discharge (from hospital discharge prescriptions). Inhaled maintenance therapy was considered to have been optimized as per the 2023 CTS COPD guidelines if prescriptions at discharge were in keeping with the recommendations from the sections <<*Preventing AECOPD*>> and <<*Reducing mortality*>>.

These subjects’ demographic and clinical characteristics were compared to those of patients treated at Montfort Hospital in 2022, before the revised 2023 CTS guidelines. The proportion of individuals receiving recommended inhaler optimization was also analyzed. The COPD care pathway in place was the same during both of the study periods, and it did not include the prescription of maintenance inhaled therapy. Student's t-test was used for continuous variables and Pearson's chi-squared test for categorical variables. Continuous results were presented as means with range and categorical results as total numbers and percentages. A statistically significant relationship was defined as P < 0.05. All statistical analyses were completed using IBM SPSS Statistics version 22 (Armonk, New York). STROBE reporting guidelines were consulted.^8^

## Results

A total of 169 unique patients were treated for AECOPD between October 2023 and September 2024. 95 subjects (56%) required admission to an inpatient unit (mean length of stay of 8 days). 3 patients (2%) died in the hospital from COPD. The mean age was 70 years old, with 90 individuals being male (53%). 90 (53%) were active smokers; the mean pack-year history was 50. 38 patients (22%) used home supplemental oxygen. Spirometry results were available for 64 subjects (38%), with a mean forced expiratory volume in the first second of 48% predicted. The mean peak BEC in the prior 12 months among all patients was 0.31 × 109/L. Demographic and clinical characteristics were similar to those of patients treated for AECOPD in 2022 (Table 1).

**Table 1.** Demographic and clinical characteristics of patients treated for a severe acute exacerbation of chronic obstructive pulmonary disease.

Characteristics	2022 (214)	October 2023 to September 2024 (169)	P-value
Admission to an inpatient unit n (%)	135 (63%)	95 (56%)	0.17
Age, years mean (SD)	70 (11)	70 (10)	0.61
Sex, male n (%)	113 (53%)	90 (53%)	0.93
Smoking status, active n (%)	109 (51%)	90 (53%)	0.65
Smoking history, pack-years mean (SD)	51 (25)	50 (20)	0.68
Home oxygen use n (%)	43 (20%)	38 (22%)	0.57
Spirometry report available n (%)	92 (43%)	64 (38%)	0.31
FEV1, % predicted (when available) mean (SD)	49 (20)	48 (19)	0.74
Peak BEC, 10^9^/L in the past 12 months mean (SD)	0.24 (0.36)	0.31 (0.40)	0.08

BEC, blood eosinophil count; FEV1, forced expiratory volume in the first second; L, litre; SD, standard deviation.

Ninety-seven patients (57%) were prescribed inhaled LAMA/LABA/ICS at the time of hospital presentation (47% with single-inhaler triple therapy (SITT)), 12 (7%) LAMA/LABA, 9 (5%) ICS/LABA, 7 (4%) LAMA monotherapy, 6 (4%) ICS monotherapy, and only 1 ICS and LAMA. 37 subjects (22%) were on no long-acting medication. SITT use significantly increased from 2022 (P<0.01) (Table 2).

**Table 2.** Inhaled triple therapy use in patients treated for a severe acute exacerbation of chronic obstructive pulmonary disease.

Inhaled therapy	2022 (214)	October 2023 to September 2024 (169)	P-value
Inhaled LAMA/LABA/ICS at admission n (%)	104 (49%)	97 (57%)	0.09
SITT at admission n (%)	20/104 (19%)	46/97 (47%)	< **0.01**

Bold indicates P-value<0.05.

ICS, inhaled corticosteroid; LABA, long-acting beta-agonist; LAMA, long-acting muscarinic antagonist; SITT, single-inhaler triple therapy.

**Figure 1.**
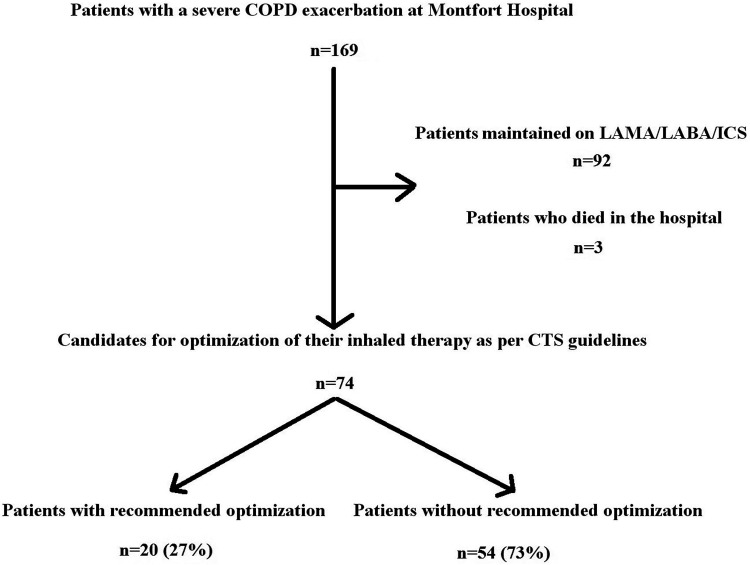
Candidates for review of their inhaled therapy.

After excluding patients who died in the hospital and those who were maintained on inhaled LAMA/LABA/ICS (92), 74 were candidates for review of their inhaled therapy. Of these, 20 (27%) received recommended pharmacotherapy (Figure 1). Adherence to the CTS COPD pharmacotherapy guidelines did not significantly improve overall since the publication of the revised recommendations (P = 0.25). Patients admitted to an inpatient unit were however more likely to receive appropriate optimization of their pharmacotherapy at discharge than in 2022 (51% vs 27%, P = 0.02). Only 5% of patients treated exclusively in the ED received recommended optimization (Table 3). The most common deviations from the CTS guidelines were continuing patients on their previous therapy (35%) and prescribing no long-acting inhaled medication (22%) (Table 4).

**Table 3.** Adherence to the Canadian Thoracic Society chronic obstructive pulmonary disease pharmacotherapy guidelines.

Care setting	2022 (111)	October 2023 to September 2024 (74)	P-value
Overall n (%)	22 (20%)	20 (27%)	0.25
Inpatient Admission n (%)	17/62 (27%)	18/35 (51%)	**0.02**
Emergency Department n (%)	5/49 (10%)	2/39 (5%)	0.38

Bold indicates P-value<0.05.

**Table 4.** Deviations from the Canadian Thoracic Society chronic obstructive pulmonary disease pharmacotherapy guidelines.

Deviations (54)	n (%)
No step-up of maintenance therapy	19 (35%)
Discharge on no maintenance therapy	12 (22%)
Step-up to LAMA/LABA rather than LAMA/LABA/ICS	7 (13%)
Step-up to ICS/LABA rather than LAMA/LABA/ICS	7 (13%)
Discharged on ICS monotherapy	6 (11%)
Step-down from LAMA/LABA/ICS	2 (4%)
Discharge on duplicate long-acting bronchodilators (same class)	1 (2%)

ICS, inhaled corticosteroid; LABA, long-acting beta-agonist; LAMA, long-acting muscarinic antagonist.

## Discussion

The 2023 CTS COPD pharmacotherapy guidelines now propose a more proactive approach for patients at high risk of AECOPD by recommending inhaled LAMA/LABA/ICS as initial maintenance therapy, preferably provided in a single inhaler. The COPD guideline committee emphasized the randomized clinical trial data showing a reduction of all-cause mortality with SITT compared to LAMA/LABA in patients with a prior history of AECOPD.^4–6^ Two different SITT options are also now approved for use in Canada. Despite this simplified treatment approach for clinicians, overall adherence to the CTS guidelines remained low in the hospital setting.

A key positive finding from this study was the improved observance of the guidelines seen among hospitalized patients. It is possible that adherence could even further increase over time with enhanced physician awareness of the revised CTS recommendations. For example, 14 subjects were prescribed inhaled dual therapy (LAMA/LABA or ICS/LABA) in accordance with the 2019 statement instead of triple therapy.^3^ Optimization of maintenance COPD therapy however remains a tremendous challenge in the ED setting. Underutilization and poor access to spirometry, excessive time constraints, and perceptions regarding the roles of physicians in the acute care setting are likely contributing factors. Healthcare resource utilization has been criticized as a measure for grading the severity of AECOPD.^9,10^ As patients can self-present to the ED, AECOPD treated in this setting could often be managed in an outpatient clinic and therefore categorized as either a mild or moderate event. Nevertheless, this situation should constitute a serious concern for our policymakers as a growing proportion of the local population does not have access to a primary care provider.^11,12^ This review suggests that structured ED-based interventions, such as the implementation of standardized COPD care plans addressing maintenance inhaled therapy, are likely necessary to significantly increase adherence to the CTS COPD guidelines. This could be achieved through physician order sets in electronic medical record systems or by involving allied health professionals such as pharmacists, nurse practitioners, and respiratory therapists.

Regardless of the care setting, the respiratory community must continue its efforts to draw attention to the importance of preventing AECOPD. These events are often life-altering for patients and should be called in layman's terms <<acute lung attacks>>. Despite being associated with significant patient morbidity and mortality, 73% of severe AECOPD patients treated at Montfort Hospital, a large academic institution, did not receive guideline-based therapy at hospital discharge.^13–20^ Such a lack of observance of the national treatment recommendations, which is likely widespread across Canada, would be considered unimaginable for most other acute disorders.^21^ It is necessary for medical professionals to consider AECOPD as an opportunity to optimize nonpharmacological and pharmacological management of the disease to prevent recurrent events.

This study has several limitations. A minority of subjects had spirometry results on file. We should consider the possibility that some individuals were misdiagnosed with COPD rather than another pulmonary condition. The retrospective nature of this review makes it difficult to determine if the initial complete blood count was done before or after systemic corticosteroid initiation, which could impact the results of BEC. The study methodology prevented assessing for a history of moderate AECOPD. Lastly, this was a single-center Canadian review and the findings may not be generalizable to all other acute care hospitals.

## Conclusion

In summary, this real-world hospital study revealed that within the first 12 months after the publication of the 2023 CTS COPD pharmacotherapy guidelines, 27% (20/74) of candidates for medication optimization received recommended therapy at discharge. While adherence to the CTS recommendations significantly increased for hospitalized patients compared to 2022, the lack of improvement seen in ED-treated subjects highlights the need for structured COPD care plans in this specific setting. Further implementation of the CTS COPD pharmacotherapy guidelines is crucial for reducing the burden of AECOPD on patients and the healthcare system.

## Supplemental Material

sj-docx-1-cra-10.1177_29768675251336660 - Supplemental material for Evaluating Adherence to the 2023 Canadian Thoracic Society Chronic Obstructive Pulmonary Disease Pharmacotherapy Guidelines: A Hospital-Based Study Supplemental material, sj-docx-1-cra-10.1177_29768675251336660 for Evaluating Adherence to the 2023 Canadian Thoracic Society Chronic Obstructive Pulmonary Disease Pharmacotherapy Guidelines: A Hospital-Based Study by Mathieu D. Saint-Pierre in Therapeutic Advances in Pulmonary and Critical Care Medicine
